# Accelerated imaging of rest and stress myocardial perfusion MRI using multi-coil k-t SLR: a feasibility study

**DOI:** 10.1186/1532-429X-14-S1-P239

**Published:** 2012-02-01

**Authors:** Sajan Goud Lingala, Edward DiBella, Mathews Jacob

**Affiliations:** 1 Biomedical Engineering, The University of Iowa, Iowa city, IA, USA; 2 Radiology, University of Utah, Salt lake city, UT, USA; 3 Electrical and Computer Engineering, The University of Iowa, Iowa city, IA, USA

## Background

In myocardial perfusion imaging (MPI), highly accelerated acquisition can be used towards reducing the compromises in the image quality (spatio-temporal resolutions, volume coverage, SNR) routinely observed with the current clinical protocols. In this study, we demonstrate the feasibility of a recent accelerated dynamic MRI scheme, k-t SLR (based on sparse and low rank properties) (Lingala et al. '11) in free breathing MPI. k-t SLR exploits natural redundancy in MPI by using (a) the high degree of temporal correlations, and (b) the sparse properties of the images in appropriate transform domains (finite difference transforms along space and time). It poses the recovery as a spectral regularization problem, allowing for the use of fast optimization solvers and flexible non-Cartesian sampling schemes. Here, we also include information from multiple coils to improve data consistency in the recovery. We demonstrate high accelerations with both rest and stress data sets. Comparisons are made with existing schemes such as spatio-temporal constrained reconstruction (STCR, Adluru et al '09) and k-t SPARSE/SENSE (Otazo et al '10).

## Methods

A radial FLASH saturation recovery sequence (TR/TE~2.5/1.3ms) was used. 3 slices with 72 rays uniformly spaced within a frame and offset between frames were acquired. Datasets for 3 normal subjects and 1 patient with disease were imaged in rest and adenosine stress. These 72 ray sets correspond to an acceleration of R ~ 3.4 compared to Nyquist sampling. Residual streaking was resolved in these sets by using a standard STCR algorithm and formed the ‘reference’ images. We performed retrospective undersampling using 21 rays (R~12) and 18 rays (R~14) respectively for the stress and rest sets and reconstructed with multi-coil k-t SLR, multi-coil STCR and multi-coil k-t SPARSE. Golden ratio, uniform and random sampling were respectively used for k-t SLR, STCR and k-t SPARSE/SENSE, based on experiments of which sampling pattern was best for each algorithm.

Validation was based on two metrics that depict the accuracy of the myocardial perfusion dynamics (see fig 2). The first metric gives a measure of the myocardial slope while the second gives an aggregate measure of several factors such as peak enhancement, sensitivity to breathing motion and temporal blur.

**Figure 1:**
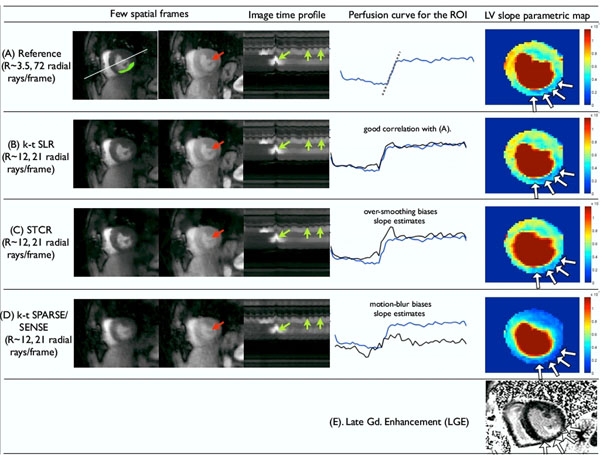
Performance comparison of different schemes using stress data of patient with myocardial infarction. Here, we show two time frames, image time profile (through a cross section in (A)), an averaged ROI perfusion curve, and parametric slope maps for the different schemes. The patient had reduced myocardial perfusion uptake in the inferior wall as depicted in the slope maps. This was later confirmed as infarction by LGE as shown in (E), where the infarct region is thinner compared to the normal myocardium. k-t SLR reconstructions depict these regions efficiently in comparison to STCR and k-t SPARSE/SENSE methods as seen in (A-D). Specifically, STCR and k-t SPARSE/SENSE show over-smoothing and motion blur respectively, while k-t SLR is robust to this as depicted by the red and green arrows in the spatial frames and the time profiles. The differences are reflected in the perfusion curves as well.

**Figure 2:**
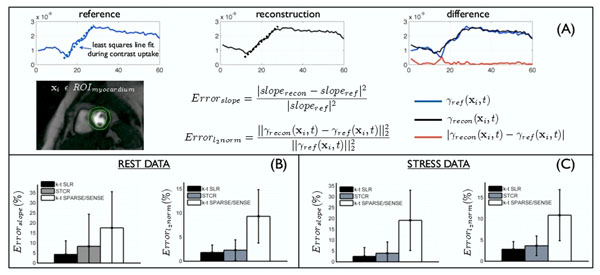
Validation using metrics of errors in the slope and the overall time curve. Initially, each reference data set was registered to estimate the deformation maps that correspond to the breathing motion. These maps were used to deform the reconstructions followed by which two error metrics were evaluated. With respect to the reference images, the error in the slope and error in the l2 norm of the pixel time series were calculated for the different reconstructions as depicted in (A). This was done for all the pixels belonging to regions of the myocardium for all the data sets. The mean +/- standard deviation of these metrics are plotted in (B) and (C). We observed consistently reduced errors with k-t SLR in comparison to STCR and k-t SPARSE/SENSE.

## Results

We observe k-t SLR to provide good correlation with the reference images. k-t SLR is robust to artifacts such as over-smoothing and motion blur, yielding better slope estimates compared to STCR and k-t SPARSE/SENSE (fig 1). In comparison to existing schemes, k-t SLR obtained significantly less error in the metrics over all the data sets (fig 2).

## Conclusions

We demonstrated high accelerations in rest and stress imaging. This can be used towards improving a number of factors such as increasing the number of slices, and performing rapid scans such as systolic imaging or ungated imaging, where the acquisition window is significantly shorter than what is usually seen in diastolic imaging.

## Funding

NSF AWARD CCF-0844812 and in part by NIH R01EB006155.

